# Anatomic and Clinical Effects of Focal Laser Ablation of the Prostate on Symptomatic Benign Prostatic Hyperplasia

**DOI:** 10.3390/cancers17030475

**Published:** 2025-01-31

**Authors:** Eric M. Walser, René Zimmerer, Anne Nance, Irfan Masood, Arsalan Saleem

**Affiliations:** Department of Radiology, The University of Texas Medical Branch, Galveston, TX 77555-0709, USA; rezimmer@utmb.edu (R.Z.); amnance@utmb.edu (A.N.); irmasood@utmb.edu (I.M.); arsaleem@utmb.edu (A.S.)

**Keywords:** laser ablation, benign prostatic hyperplasia, prostate cancer

## Abstract

Background/Objectives: Laser ablation is a promising technique for tissue-debulking in patients with symptomatic benign prostatic hyperplasia (BPH). This study evaluated the effects of focused laser ablation of the prostate (FLA) on urinary symptoms for patients with BPH. Methods: Since 2018, 62 patients had bilateral prostate FLA for prostate cancer and/or symptomatic BPH, defined as an international prostate symptom score (IPSS) ≥11, and have 6-month follow-up data. Urinary and sexual health were scored with standardized surveys while imaging defined prostate anatomy. FLA was performed as an outpatient procedure with either transrectal MRI-guided (*n* = 24) or transperineal ultrasound-guided (*n* = 38) laser fiber placement to debulk the prostate and/or ablate cancer foci plus margins. Enhanced prostate MRI was performed immediately or up to 2 days later to assess the treatment zones. Follow-up then consisted of PSA levels every 6 months and MRI at 6–12 months and then yearly combined with patient sexual/urinary surveys and clinical assessments. Results: All patients had technically successful FLA and 6-month clinical and imaging follow-up. At 6-month follow-up, mean IPSS was reduced by 43% relative to baseline (10.4 vs. 18.4), mean prostate volume was reduced by 30% (42.2 vs. 60.5 mL), and mean PSA was reduced by 58% (4.3 vs. 10.2 ng/mL). All of these changes were statistically significant (*p* ≤ 0.008). Compared with baseline, there was no significant change in the SHIM score at 6 months (16.0 vs. 16.8; *p* = 0.59). In a subset of patients for whom 12-month data were available, there were significant reductions in PSA (61%; 4.1 vs. 10.5 ng/mL; *p* < 0.002) and IPSS (45%; 9.9 vs. 17.9; *p* < 0.002), while the 12-month SHIM score was not significantly different from baseline (15.2 vs. 16.0; *p* = 0.27). Mean laser irradiation time was 19 min with a mean energy deposition of 13,562 J. The most frequent adverse events were prolonged urinary catheterization in 10 patients (16%) and urinary tract infection in 8 (13%). Conclusions: FLA is a safe and effective tissue-debulking technique for patients with symptomatic BPH. This outpatient procedure requires minimal procedure time and can be performed without the need for operating rooms or cystoscopy. Our results are consistent with those of previous studies indicating that FLA preserves sexual function.

## Simple Summary

Benign prostatic hyperplasia (BPH) affects 50% of men over the age of 50, increasing to 80% of those over 80 years. While behavioral or medical therapy is effective in at least half of symptomatic cases, surgical treatments should be considered in patients refractory to conservative care. Earlier definitive treatment is also supported by studies revealing the side effects of medications taken chronically for BPH, including dementia, depression, and decreased sexual drive and function. While a host of minimally invasive cystoscopically directed surgical treatments (MISTs) for BPH are available, transrectal or transperineal laser ablation for debulking BPH is emerging as a safe, effective, and economical alternative. This study looks at the urinary benefits of laser prostate ablation weighed against any negative side effects. If laser ablation proves safe and effective, the impact on men’s health is significant as the procedure is inexpensive, repeatable, and independent of an operating room environment or expensive cystoscopic apparatus.

## 1. Introduction

Benign prostatic hyperplasia (BPH) is the most common prostatic disease, becoming more prevalent with advancing age, and affecting 70% of men aged 60−69 years [1]. While prostatectomy is still a viable treatment option for BPH, it is rarely considered except in the most extreme cases of prostate enlargement with intravesical extension. Currently, the vast majority of BPH therapies utilize minimally invasive surgical techniques (MISTs) for BPH and all rely on cystoscopic fulguration of periurethral tissue or expansion of the urethral channel to improve urinary function. The gold standard of MISTs is still considered to be the transurethral resection of prostate tissue (TURP) [2,3]; other MISTs include robotic water jet treatment (Aquablation), prostatic urethral lift (UroLift), convective water vapor thermal therapy (Rezūm), and temporary implantable nitinol devices [4]. MISTs, however, require a facility and equipment for cystoscopy and may cause troubling side effects such as retrograde ejaculation, bleeding, fluid overload, and erectile dysfunction [4].

Focal laser ablation of the prostate (FLA) is an alternative technique derived from other image-guided percutaneous laser ablative therapies for conditions such as hepatocellular carcinoma, thyroid carcinoma, and benign thyroid nodules [5]. The procedure does not require cystoscopy and delivers energy via a transrectal or transperineal approach. FLA is described in several European case series indicating that it is a safe approach to treating lower urinary tract obstructive symptoms (LUTSs) due to BPH [6,7], with an average reduction in prostate volume of greater than 30% at one year with significant improvement of symptoms and quality of life at 6 and 12 months post-procedure [8]. More recent studies have reported the durable benefit of FLA at 3-year follow-up [9] and beyond (median follow-up of 56.5 months) [10]. The promising early results of this minimally invasive technology suggest a safe, effective, and inexpensive alternative approach to surgery or prostate artery embolization (PAE) for symptomatic BPH.

The aim of this retrospective study was to evaluate the efficacy and safety of prostate FLA in a cohort of 62 patients with prostate cancer and BPH, or BPH alone. The study demonstrates that FLA is a safe outpatient procedure that is suitable for treating both prostate cancer and symptomatic BPH in a single session, producing significant improvements in urinary symptoms and prostate volume, while preserving sexual health.

## 2. Materials and Methods

Between 2018 and August 2024, 349 patients underwent prostate FLA for either low- or intermediate-risk prostate cancer, symptomatic BPH defined as an International Prostate Symptom Severity (IPSS) score of 11 or greater, or both conditions. Of these patients, 62 had bilateral FLA for prostate cancer and BPH or BPH alone and have complete data at 6-month follow-up. A total of 24 patients had transrectal FLA using MRI guidance and real-time MR thermometry and 38 patients had transperineal FLA with transrectal ultrasound guidance. All patients had bilateral ablations, and 48 patients were also treated for focal prostate cancers while 14 patients were treated exclusively for BPH. No patients had indwelling urinary catheters, and all cancer patients were Gleason 7 or less with disease confined to the prostate. Exclusion criteria for offering a transperineal or transrectal procedure were uncorrectable coagulopathy, surgically or congenitally absent anus/rectum, and BPH with a predominant intravesical component (type 4 or 5 BPH) [11]. The diagnosis of BPH was made on clinical grounds, including an IPSS score >10 and prostate MRI-documented gland size and configuration in all cases. Sexual and urinary symptoms were captured with Sexual Health in Men (SHIM) and IPSS surveys [12,13]. Urodynamics data are gathered for our current patients but were not obtained consistently in this early series of patients.

All FLA procedures were performed by a single operator with over 20 years’ experience in ablative therapies (E.M.W.). All patients received oral antibiotics (cefdinir or ciprofloxacin) on the day before the procedure, and this continued for 5 days. Patients self-administered enemas the day before and the morning of the procedure. An intravenous dose of antibiotics (cefazolin 1–2 g) was administered during the procedure. Patients with a history of post-prostate-procedure sepsis or ciprofloxacin-resistant *E. coli* colonization received intravenous meropenam during the procedure. Patients received intravenous moderate sedation during the procedures.

**Figure 1:**
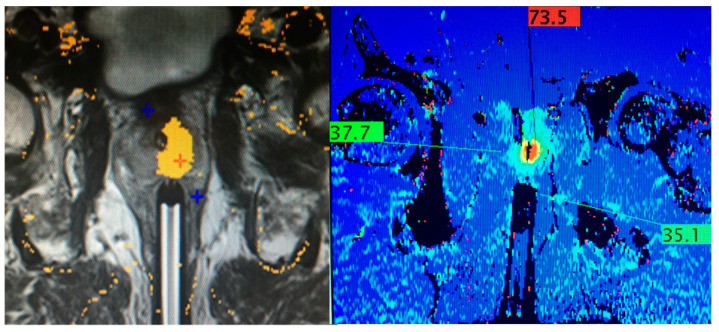
Thermal imaging during MRI-guided transrectal laser ablation of the left gland. (**Left image**): the yellow zone indicates irreversible tissue damage. Notice sparing of the central urethra due to cooling saline infusion through the indwelling Foley catheter. (**Right image**): real-time thermometry with electronic calipers indicating estimated temperatures during laser ablation. Above 60 °C, there is instant cell death. The lower right caliper monitors rectal wall temperature, shown as 35 °C (normal).

For MRI, we performed FLA with a transrectal approach, using one saline-cooled fiber to create overlapping ablations through a rectal probe. The laser fiber and the rectal probe were guided to the prostate cancer lesions using the DynaTRIM (Transrectal Interventional MRI) system (Philips, Amsterdam, The Netherlands), which gives the rectal probe three planes of position adjustment to guide the laser using MR images in any plane. The FLA procedure involved the Visualase thermal therapy system (Medtronic, Minneapolis, MN, USA) comprising a computer workstation, a 30 W 980 nm diode laser, a cooling pump, and a (disposable) laser applicator set composed of a 600 µm core silica fiber optic with a cylindrical diffusing tip housed within a 1.85 mm diameter saline-cooled polycarbonate cooling catheter. Extracted thermal data produce color-coded ‘thermal’ and ‘damage’ images based on an Arrhenius rate process model which are displayed on the workstation. The damage image accounts for the cumulative effects of the time–temperature history of each voxel in the image. The diffusing laser fiber tip creates a 16–18 mm oval ablation zone, and the laser can be slid back and forth within the cooling cannula to ‘paint’ an ablation zone from the front to the back of the prostate gland (Figure 1). Each ablation was approximately 15 W power at 2–3 min per ablation. If the targeted lesion was close to the rectal wall, a sheathed needle was punctured into the rectoprostatic space (also transrectal) and used to hydrodissect the rectum away from the prostatic capsule using an infusion of normal saline to create a space of at least 1 cm. The growing damage estimate was watched closely to protect the neurovascular bundles and rectal wall from thermal injury [14,15,16,17]. After satisfactory ablation of prostate lesions, both prostate lobe transition zones received further ablations to relieve symptoms of BPH. Additional ablations were not performed in cancer patients without bladder obstructive symptoms.

**Figure 2:**
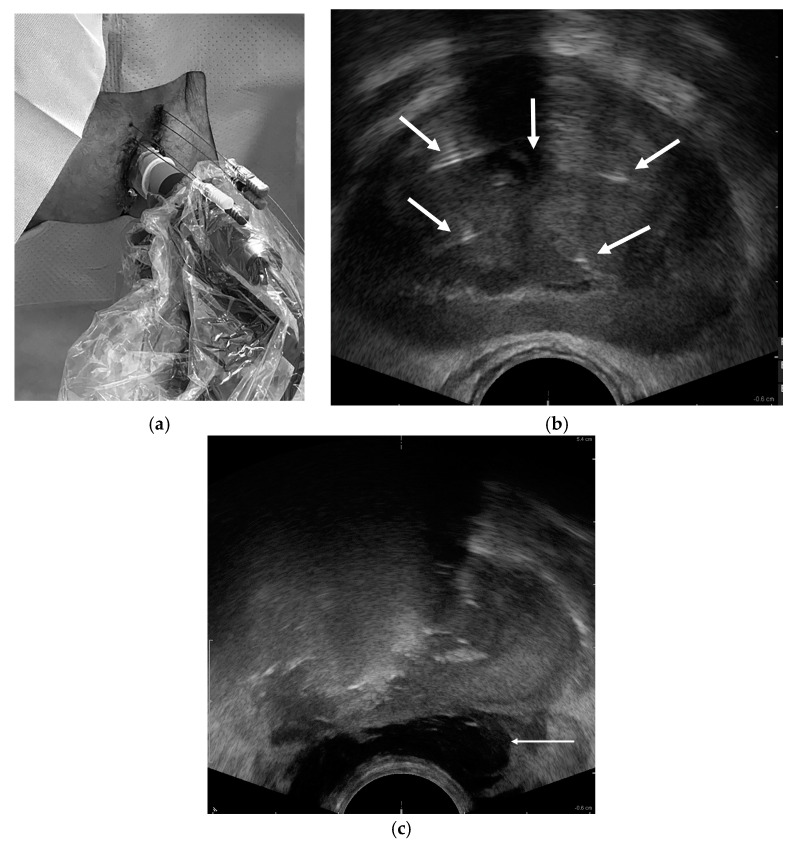
EchoLaser TPLA^TM^ (transperineal FLA) for BPH. (**a**) Focused placement of four 21 g needles using transrectal ultrasound guidance. The ultrasound probe is attached to a stabilizer (stepper) to enable stable guidance during needle placement. (**b**) Axial transrectal ultrasound image shows shadowing from Foley catheter centrally (middle arrow) and 4 periurethral needles for laser fiber positioning and ablation. (**c**) Right gland shadowing during active laser ablation and hydrodissection fluid injection in rectoprostatic space (arrow).

**Figure 3:**
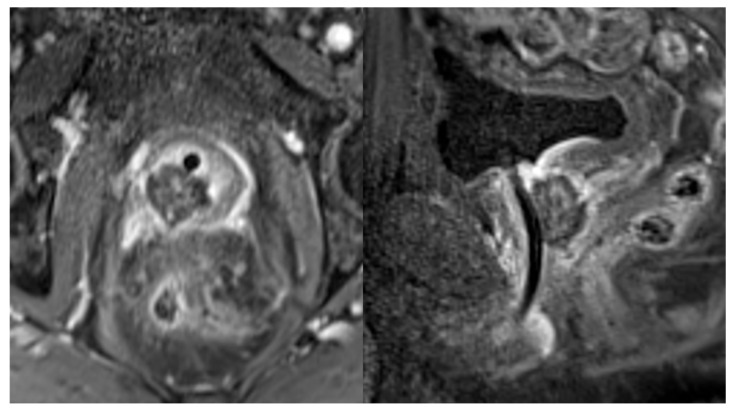
Avascular ablation zone near right prostate apex 48 h after FLA for small prostate cancer. Axial (**left**) and sagittal (**right**) T1 post-contrast images. Notice no evidence of thermal damage to anterior rectal wall. Left periurethral ablation zone is not visible in this image.

For transperineal ablations (EchoLaser TPLA^TM^), patients were positioned on a gurney with stirrups to expose the perineum. A transrectal ultrasound probe (BK Medical, Burlington, MA, USA) was inserted and attached to a ‘stepper’ device to allow real-time visualization of the prostate in the sagittal and axial orientation. Four to nine 21 g needles were placed 1 cm apart to debulk the periurethral transition-zone tissue and target prostate cancers (Figure 2). For pain control, prostate blocks were performed bilaterally with 2% lidocaine supplemented by IV moderate sedation. After positioning the needles as above, the EchoLaser X4 device (multisource laser system, Elesta S.p.A., Calenzano, Florence, Italy) enabled placement of 300 µm fiber optics (Elesta S.p.A., Calenzano, Florence, Italy) for laser ablation with a 1064 nm wavelength laser source just outside the Introducer needle (Elesta S.p.A., Calenzano, Florence, Italy) for creation of an approximately 2 cm oval ablation zone in front of the fiber tip, which was pulled back for additional ablations in larger prostates, as needed. The use of multiple fibers that work simultaneously allows an amplification of ablation volume and the concurrent treatment of the prostatic lobes [18,19]. Complete ablation occurred after deposition of 1800 J per fiber per irradiation. Each irradiation with 4 fibers deposited approximately 7200 J over 10 min. For both procedures, a 3-way Foley bladder catheter was inserted and chilled saline infused through it to prevent thermal damage to these structures (chilled urethral saline protection or CUSP). Similar to transrectal ablations, when the target lesion was near or abutting the posterior prostate capsule, a transperineal hydrodissection needle was positioned between the rectum and prostate to infuse saline and protect the neurovascular bundles and anterior rectal wall from thermal damage. A fiber distance to the capsule of ≤1 cm prompted hydrodissection due to the ablation diameter of 2 cm centered at the laser tip. Finally, all patients had a contrast-enhanced prostate MRI immediately (MRI-guided) or 48 h post-procedure (transperineal US-guided) to assess the ablation zones (Figure 3). A detailed description of the TPLA procedure has been published recently [20].

Technical success was defined as deployment of laser fibers within the prostate and completion of the prescribed energy deposition. IPSS, SHIM, serum PSA levels, and prostate volumes were measured prior to ablation and at 6 months post-procedure. Two-tailed *t* tests were performed using data functions in Microsoft^®^ Excel (Microsoft, Redmond, WA, USA) to compare these patient variables before and 6 months after ablation, with *p* < 0.05 considered statistically significant.

## 3. Results

**Figure 4:**
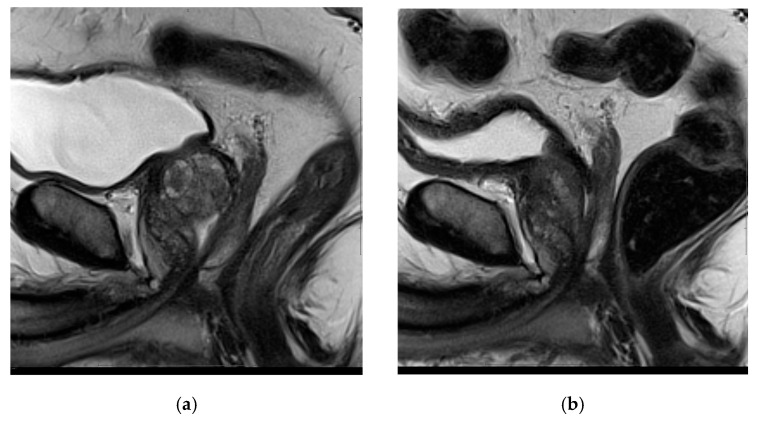
Sagittal T2 image of the prostate before (**a**) and 6 months after (**b**) bilateral FLA for BPH. Notice glandular volume reduction.

Mean ± standard deviation (SD) patient age was 66.9 ± 6.9 years. Technical success was achieved in all patients. The mean laser time was 18.7 ± 14 min with the mean energy delivered per patient being 13,562 ± 16,194 J. At 6 months follow-up, SHIM was not significantly different when compared with baseline in all groups, meaning that patients generally maintained sexual function after FLA. Prior to FLA, SHIM was 16.8 (SD 7.7), and it was 16.0 (SD 7.8) 6 months later (*p* = 0.59). PSA levels, IPSS scores, and prostate volumes were reduced significantly at mean follow-up (Table 1). Changes in these parameters include an overall 58% reduction in PSA value (4.3 vs. 10.2 ng/mL), a 30% decrease in prostate volume (42.2 vs. 60.5 mL) (Figure 4), and significant relief of bladder obstructive symptoms (IPSS was reduced from 18.4 to 10.4, a 43% reduction). All decreases were statistically significant (*p* ≤ 0.008). In a subset of 53 patients (mean age: 67.0 ± 7.0 years), 12-month follow-up data were also available. In these patients, there were statistically significant reductions in PSA and IPSS at 12 months vs. baseline (61% and 45%, respectively; both *p* < 0.002), while the 12-month SHIM score was not significantly different vs. baseline (*p* = 0.27) (Table 2). Adverse events were generally mild (Clavien–Dindo/urology grade I–II) and included 10 patients (16%) with temporary urinary retention, defined as bladder catheterization lasting >10 days, and 8 patients (13%) with confirmed urinary tract infection.

## 4. Discussion

Thermal ablation for BPH is an alternative to cystoscopically directed transurethral tissue extirpation (TURP, Holmium laser, robotic waterjet and water vapor ablations, focused ultrasound) or mechanical/stent technologies [21] or embolization procedures (PAE). FLA is one such outpatient option and enables the precise placement of one or more needles to deliver laser fibers which create reliable zones of ablation based on standardized energy deposition. We used both MRI-guided ablations with thermal imaging and transperineal ablations with transrectal ultrasound guidance. Our current preference is transperineal ablation due to the ability to place up to four needle/laser units for a single treatment, the ease of using ultrasound rather than the need to use valuable MRI time, the avoidance of infectious complications from transrectal needle placement, and shorter procedure time. Moreover, the TPLA under ultrasound guidance is supported by the use of a tool device (Echolaser Smart Interface device, Elesta S.p.A., Calenzano, Italy) that can be connected to a general ultrasound scanner and help in the positioning of the needles/fibers inside the prostatic tissue, taking into account the safety distance from critical structures [8,22].

FLA is a method to treat symptomatic BPH but has expanded indications to include focal prostate cancers using cognitive or true fusion of the prostate MRI with the transrectal ultrasound imaging [19,23,24]. With larger prostate glands or bilateral lesions, we used up to nine fibers and ablated each lobe separately using methods to keep shadowing ablated tissue out of the ultrasound path of subsequent ablations (i.e., treating the right then left lobe or treating from the anterior to posterior gland). Thermal protection of the urethra and rectum was accomplished by infusing cool saline through an irrigation Foley bladder catheter and by hydrodissecting the space between the rectum and prostate. There were no incidences of rectal burn or fistulae; however, 16% of patients experienced urothelial damage and edema, likely due to insufficient cooling via the Foley catheter, and are part of the group requiring prolonged urinary catheterization. This urethral damage was documented by retrograde urethrography showing expanded, irregular prostatic urothelium and reflux of contrast into the vas deferens. Admittedly, Foley catheters do not function well as cooling devices due to thick construction and insufficient radial coverage of the catheter by internal fluid flow. We are moving towards better urethral cooling catheters as we continue our experience with FLA. Minor complaints were primarily related to the consequences of chronic bladder catheterization (e.g., discomfort, social unease, inability to have normal sexual relations), and patients did not experience significant post-procedure pain and did not require oral narcotics.

With an increasing number of studies of TPLA for BPH, an overview of the potential adverse events of the procedure is possible. Apart from the cases of prolonged catheterization and urinary tract infection that we observed, hematuria may occur more frequently in patients taking antiplatelet agents or anticoagulants. However, it is usually mild, with spontaneous resolution within several days. Prostatic abscess has been reported with an incidence of 0–5% across different studies. Ejaculatory dysfunction, characterized by reduced ejaculate volume or retrograde ejaculation, has been reported in 0–4% of patients. Erectile dysfunction, urethral stricture, and incontinence have proved to be extremely rare adverse events after TPLA if the recommended safety distances are respected [20].

We judged the efficacy of ablation with an immediate post-procedure or 48 h contrasted MRI to assess the ablation zone adequacy for either treating cancer lesions plus a margin or debulking a significant portion of the periurethral BPH tissue. This method was recently shown to correlate well with observed necrosis on explanted and laser-treated prostate glands [25]. Thereafter, we followed patients’ PSA levels, expecting about a 50% reduction in 6 months. Yearly MRI and PSA trends were used to follow the patients with concomitant low- to intermediate-risk prostate cancer. Developing MRI lesions and/or increasing PSA levels prompted biopsy and consideration of further treatment. Several other investigators are reporting their results using FLA for BPH and, less commonly, prostate cancer. Manenti et al. recently reported 44 patients who had FLA for BPH and observed a 54% decrease in prostate volume and significant improvement in symptoms of lower urinary tract obstruction. The authors noted a lower incidence of urinary retention (11.3%) and an overall adverse event rate of 7% [26]. A systematic review of FLA for BPH, published by Tafuri et al. in 2023, showed significant improvements in urinary flow, postvoid residual, and IPSS score in almost 300 patients in pooled studies with no concomitant effect on ejaculatory or sexual function [18]. Laganà et al. reported a prospective, single-center study of TPLA in 63 BPH patients (mean prostate volume: 63.6 ± 29.7 mL). At 12 months, statistically significant improvements in IPSS, QoL, postvoid residual, and Q_max_ were observed relative to baseline, with improvements already apparent at 3 months. At 12 months, there was a relative improvement in each of the efficacy outcomes vs. 3- month results. At 12-month follow-up, all patients had discontinued medical therapy for BPH, and mean PSA was reduced from 4.82 ± 1.8 ng/mL to 2.89 ± 1.2 ng/mL. TPLA was well tolerated, with transient complications consisting of two patients with prostatic abscesses (3.2%) that were drained successfully, and one patient with orchitis (1.6%) that was successfully treated with antibiotics [27].

Data comparing TPLA directly with other treatments for BPH are limited. Two randomized, controlled trials of TPLA vs. TURP have been reported [22,28]. Bertolo et al. [22] evaluated changes in ejaculatory function as the primary endpoint in 51 patients, observing a significantly higher proportion of patients with preserved ejaculatory function in those randomized to TPLA vs. TURP (96.2% vs. 28%; *p* < 0.001). Canat et al. [28] evaluated 50 patients randomized to TPLA or TURP and reported that Male Sexual Health Questionnaire—Ejaculatory function domain scores in TPLA patients did not significantly worsen at 1-year follow-up, in contrast to significant worsening of these measures in TURP patients. In both trials, significant improvements in measures of bladder function and symptoms were observed with both techniques, although the post-procedural improvements in Q_max_ were higher with TURP as compared to TPLA [22,28]. In addition, preliminary results from two comparisons of TPLA with water vapor thermal therapy (Rezūm) have been reported—one a prospective, real-world comparison [29], the other a single-center experience from a randomized, clinical trial [30]. These initial comparisons suggest similar effects of the two treatments, including preservation of ejaculatory function.

There are multiple advantages to treating benign and malignant prostate disease with focused approaches coupled to transrectal ultrasound guidance. First, the procedures are performed in real time, with no radiation exposure to the operator or patient. Previous experience with MRI-guided laser prostate ablation included a few instances of rectourethral fistulae which probably occurred in the MRI bore when the patient moved during ablation [31]. Motion and hydrodissection are very easy to monitor and control when following the ablation progress with continuous ultrasound imaging. Second, after gaining experience in prostate needle positioning using transrectal ultrasound guidance, FLA is a relatively fast, outpatient procedure with a mean laser activation time of about 15 min. The majority of procedural time for FLA is patient preparation and accurate needle placement. Third, the FLA procedure can be tailored to treat both BPH and low- to intermediate-risk prostate cancer simultaneously, and, finally, transrectal ultrasound prostate biopsy can be performed concurrently with ablation procedures. These factors make FLA an advantageous method to treat prostate disease as compared to PAE, which takes much longer, requires radiation exposure, risks ischemic complications, and does not allow effective treatment of cancer nor biopsy of it. Efficacy and sexual side effects of the two procedures are similar [32], yet FLA can be repeated more easily than PAE due to the limitations of repeated organ embolization. PAE results in a similar reduction in IPSS scores (12–16 points) compared with our group, where IPSS was reduced from 18 at baseline to 10 at 6 months [33,34,35]. Van Riel and colleagues also reported that FLA does not compromise subsequent radical prostatectomy based on urologist surveys [19,36]. Thus, as opposed to surgery and radiation therapy, FLA does not ‘burn bridges’ to other treatments for cancer or BPH.

This study has certain strengths and limitations. This dataset expands the limited experience of the use of FLA in men with prostate cancer, and to our knowledge, is the first to report the use of the technique in men with both prostate cancer and BPH. On the other hand, the study was retrospective in nature, the number of patients with each type of prostatic disease was relatively small, and the 6-month follow-up period might not capture long-term efficacy or complications. Furthermore, there were no control groups, e.g., TURP for patients with BPH, or radical prostatectomy for patients with prostate cancer. A formal cost-effectiveness analysis was not performed; however, it is likely that TPLA compares favorably on cost with respect to MRI-based FLA, TURP, or radical prostatectomy. While studies related to the economic impact of TPLA are scarce [36], one study found that the perioperative costs of TPLA were lower than those for radical prostatectomy, TURP, HoLEP, and Greenlight Photoselective Vaporization of the Prostate, even when the cost of leasing the TPLA device was included in the calculation (EUR 2496 vs. EUR 6406 vs. EUR 4804 vs. EUR 3194 vs. EUR 4090, respectively) [37]. Compared with several of the alternative techniques, TPLA has the advantages of being capable of being performed on an outpatient basis, and without the need for cystoscopy or access to an operating room. This is likely to have a favorable effect on healthcare resource utilization.

## 5. Conclusions

FLA is a safe outpatient procedure involving no radiation or need for vascular access and allows successful debulking of BPH with excellent urinary symptom improvement, an average 30% reduction in prostate volume, and rare sexual side effects. Ablation extent can be assessed with contrast-enhanced MRI. FLA can address both cancer and symptomatic BPH in a single session and does not hinder future surgical or radiation treatments.

## Figures and Tables

**Table 1:** Prostate data before and 6 months after FLA.

Variable	Timepoint		Change (%)	*p*-Value
	Baseline	6 Months Post-Procedure		
PSA (ng/mL), mean ± SD	10.2 ± 8.9	4.3 ± 3.4	–58	<0.002
Prostate volume (mL), mean ± SD	60.5 ± 42.5	42.2 ± 27.1	–30	0.008
IPSS, mean ± SD	18.4 ± 6.5	10.4 ± 6.4	–43	<0.002
SHIM, mean ± SD	16.8 ± 7.7	16.0 ± 7.8	<5	0.59

IPSS, International Prostate Symptom Score; PSA, prostate-specific antigen; SD, standard deviation; SHIM, Sexual Health Inventory for Men questionnaire.

**Table 2:** Prostate data before and after FLA in patients with 12-month follow-up.

Variable	Timepoint		Change (%)	*p*-Value
	Baseline	12 Months Post-Procedure		
PSA (ng/mL), mean ± SD (*n* = 53)	10.5 ± 9.8	4.1 ± 3.0	–61	<0.002
IPSS, mean ± SD (*n* = 53)	17.9 ± 7.0	9.9 ± 6.8	–45	<0.002
SHIM, mean ± SD (*n* = 53)	16.0 ± 7.9	15.2 ± 7.4	<5	0.27

IPSS, International Prostate Symptom Score; PSA, prostate-specific antigen; SD, standard deviation; SHIM, Sexual Health Inventory for Men questionnaire.

## Data Availability

The data presented in this study are available on request from the corresponding author due to protected health information in patient medical records.
